# The Impact of Preoperative Dexamethasone on the Magnitude of the Postoperative Systemic Inflammatory Response and Complications Following Surgery for Colorectal Cancer

**DOI:** 10.1245/s10434-017-5817-3

**Published:** 2017-03-01

**Authors:** Stephen T. McSorley, Campbell S. D. Roxburgh, Paul G. Horgan, Donald C. McMillan

**Affiliations:** Academic Unit of Surgery, School of Medicine, University of Glasgow, Glasgow, Scotland, UK

## Abstract

**Background:**

The magnitude of the postoperative systemic inflammatory response (SIR), as evidenced by C-reactive protein (CRP), is associated with both short- and long-term outcomes following surgery for colorectal cancer. The present study examined the impact of preoperative dexamethasone on the postoperative SIR and complications following elective surgery for colorectal cancer.

**Methods:**

Patients who underwent elective surgery, with curative intent, for colorectal cancer at a single center between 2008 and 2016 were included (*n* = 556) in this study. Data on the use of preoperative dexamethasone were obtained from anesthetic records, and its impact on CRP on postoperative days (PODs) 3 and 4, as well as postoperative complications, was assessed using propensity score matching (*n* = 276).

**Results:**

In the propensity score-matched cohort, preoperative dexamethasone was associated with fewer patients exceeding the established CRP threshold of 150 mg/L on POD 3 (odds ratio [OR] 0.42, 95% confidence interval [CI] 0.26–0.70, *p* < 0.001) and fewer postoperative complications (OR 0.53, 95% CI 0.33–0.86, *p* = 0.009). Similar results for both POD 3 CRP and complications were observed when using propensity score-adjusted regression (OR 0.40, 95% CI 0.28–0.57 and OR 0.57, 95% CI 0.41–0.80, respectively) and propensity score stratification (OR 0.41, 95% CI 0.25–0.57 and OR 0.53, 95% CI 0.33–0.86, respectively).

**Conclusions:**

Preoperative dexamethasone was associated with a lower postoperative SIR and fewer complications following elective surgery for colorectal cancer.

Colorectal cancer is a leading cause of mortality in the developed world.^1^ Surgical resection continues to form the cornerstone of its management but is itself associated with significant morbidity and mortality.^2^ Long-term outcome is primarily related to disease stage at presentation, however it is increasingly understood that postoperative complications have a negative impact on oncologic outcome.^3^,^4^ In addition, postoperative complications are associated with a significant healthcare and societal cost.

Interleukin (IL)-6 and the more routinely available C-reactive protein (CRP), measured in the postoperative period, have been reported to be reliable markers of the magnitude of the surgical trauma.^5^ Furthermore, an association between the magnitude of this postoperative systemic inflammatory response (SIR) and the development of postoperative infective complications has been reported following surgery for colorectal cancer^6^–^8^ independent of presentation,^9^ and surgical approach.^10^ In addition, postoperative CRP and the threshold concentrations established to predict the development of infective complications have been reported to be associated with complication severity.^11^,^12^ More recently, a comprehensive systematic review suggested that CRP concentrations >150 mg/L on postoperative days (PODs) 3–5 should prompt investigation of potential postoperative complications such as anastomotic leak.^13^

There is good evidence that, compared with open surgery, laparoscopic surgery is associated with a reduction in the postoperative SIR.^5^ However, no definite causal relationship has yet been defined between attenuation of the postoperative SIR and postoperative complications. Furthermore, it remains to be seen whether strategies that attenuate the postoperative SIR may also reduce postoperative complication rates.

Corticosteroids, administered at the induction of anesthesia, are associated with the prevention of postoperative nausea and vomiting.^14^ Indeed, preoperative dexamethasone has now been integrated into many ‘enhanced recovery’ and ‘fast track’ perioperative care protocols, although the underlying mechanism remains unclear.^15^ Furthermore, there is evidence that preoperative administration of corticosteroids is associated with a reduction in the postoperative SIR following abdominal surgery.^16^,^17^

The aim of the present study was to examine the impact of preoperative dexamethasone on the magnitude of the postoperative SIR and complications following surgery for colorectal cancer. A propensity score analysis was performed due to significant imbalances in patient and operative variables potentially associated with both the postoperative SIR and complications.

## Patients and Methods

### Patients

This retrospective observational study of a prospectively collected database included patients who underwent resection with curative intent for histologically confirmed colorectal cancer in a single center between 2008 and 2016. Patients without available anesthetic records, receiving long-term corticosteroids, and who had existing inflammatory conditions, emergency surgery, or metastatic disease were not included in the analysis.

Clinical, radiological, and pathological data of all patients were reviewed by a specialist colorectal oncology multidisciplinary team before and after surgery. All patients received prophylactic antibiotics and venous thromboprophylaxis prior to the induction of anesthesia as per hospital policy. Furthermore, all patients were cared for in line with a unit standardized perioperative care policy that included early postoperative mobilization, early enteral nutrition, and the avoidance of routine nasogastric or peritoneal drainage. The use of epidural anesthesia was at the discretion of the anesthetic and surgical teams. Patients were administered dexamethasone intravenously prior to the induction of anesthesia, and at the discretion of the anesthetist, to reduce the likelihood of postoperative nausea and vomiting.

On each POD patients were clinically assessed and had blood samples, including serum CRP, obtained as standard until discharged. Further postoperative investigation and intervention was at the discretion of the patient’s surgical team who were not blind to serum CRP results.

### Methods

Clinicopathological data were collected prospectively in a database, anonymized, and subsequently analyzed. Recorded information included patient demographics, tumor site, TNM stage (TNM, 5th edition, American Joint Committeee on Cancer), surgical approach, complications, and preoperative and postoperative serum CRP measurements.

Serum concentrations of CRP (mg/L) were measured using an autoanalyzer (Architect; Abbot Diagnostics, Maidenhead, UK) with a lower detectable limit of 0.2 mg/L, as was serum albumin (normal range 35–50 g/L). Exceeding the established CRP threshold of 150 mg/L on PODs 3 or 4 was recorded.^13^ The preoperative modified Glasgow Prognostic Score (mGPS) was calculated in patients for whom preoperative serum CRP and albumin were available.^18^

Data regarding the use of dexamethasone for the prevention of postoperative nausea and vomiting at the induction of anesthesia, the use of epidural anesthesia, and the need for intraoperative blood transfusion were collected by retrospective review of anesthetic notes.

Complications were recorded and categorized by severity using the Clavien–Dindo scale.^19^ Infective complications were categorized as described elsewhere and briefly summarized here.^6^ Wound (superficial surgical site) infection was defined as the presence of pus either spontaneously discharging from the wound or requiring drainage; deep surgical site infection was defined as surgical or image-guided drainage of intra-abdominal pus; anastomotic leak was defined as radiologically verified fistula to bowel anastomosis or diagnosed at laparotomy; pneumonia was defined by fever above 38.5 °C and consolidatory chest X-ray findings requiring antibiotic treatment; and septicemia was defined by the presence of sepsis combined with positive blood culture. Urinary tract infection was only included if complicated by septicemia and confirmed with positive urine culture.

This study was approved by the West of Scotland Research Ethics Committee, Glasgow, as part of a surgical audit.

### Statistical Analysis

In the initial unmatched cohort, categorical data were compared using the *χ* ^2^ test. Data regarding postoperative CRP were non-normal and are presented as medians and ranges, and the medians of the two groups were compared using the Mann–Whitney *U* test. The treatment effect of preoperative dexamethasone in terms of exceeding the postoperative CRP threshold and complications was displayed as odds ratios (ORs) and 95% confidence intervals (CIs), while the magnitude of CRP by each POD was displayed graphically as 95% CIs of the median.

Multivariate logistic regression was used to generate a propensity score for each patient, predicting the probability of having received preoperative dexamethasone or not, based on the following variables thought to be associated with the postoperative SIR or complications: age, sex, body mass index (BMI), smoking status, American Society of Anesthesiologists (ASA) score, mGPS, tumor site, TNM stage, neoadjuvant chemoradiotherapy, surgical approach (open or laparoscopic), operation duration, blood transfusion, stoma formation, and the use of epidural anesthesia. Patients who received preoperative dexamethasone were then matched 1:1 with a patient who did not, using the closest propensity score on the logit scale (calliper < 0.05, order of match selection randomized, without replacement). Categorical data were compared using McNemar’s test, and continuous data were compared using the related samples Wilcoxon sign-rank test. The appropriateness of the propensity score matching was assessed visually by frequency of propensity scores in each group before and after matching. In addition, the propensity scores were included as a linear covariate alongside preoperative dexamethasone in multivariate binary logistic regression models for exceeding the POD 3 CRP threshold and postoperative complications. Finally, the propensity scores were used to stratify the patients by quintiles, from which an average treatment effect was calculated for both the POD 3 CRP threshold and postoperative complications as an OR and 95% CI.

In all tests, a two-sided *p* value <0.05 was considered statistically significant. Propensity scoring and matching, as well as all statistical analyses, were performed using IBM SPSS version 21 for Windows (IBM Corporation, Armonk, NY, USA).

## Results

### Patient Characteristics

Overall, 556 patients were included in the study (Table 1), of whom 310 were male (56%) and 360 (65%) were over 65 years of age. Most had colonic (355, 64%) and node-negative disease (375, 67%). Laparoscopic resection was performed in 212 patients (38%), with the remainder having open surgery. A postoperative complication occurred in 234 cases (42%), of which 151 (27%) were infective and 47 (8%) were classified as Clavien–Dindo grade 3–5 severity. Anastomotic leak occurred in 19 cases (3%), and there were 5 (1%) postoperative deaths.

**Table 1:** Association between clinicopathological characteristics, perioperative factors, and preoperative dexamethasone in patients undergoing any surgery for colorectal cancer [*n* = 556]

Characteristic	All [*n* (%)]	Preoperative dexamethasone		*p* value
		No *n* (%)	Yes *n* (%)	
*n*	556 (100)	245 (44)	311 (56)	–
Age, years				
<65	196 (35)	85 (35)	111 (36)	0.214
65–74	219 (40)	88 (36)	131 (42)	
>74	141 (25)	72 (29)	69 (22)	
Sex				
Male	310 (56)	139 (57)	171 (55)	0.731
Female	246 (44)	106 (43)	140 (45)	
BMI, kg/m^2^				
<20	38 (7)	14 (6)	24 (8)	0.242
20–25	170 (32)	74 (32)	96 (31)	
26–30	172 (32)	65 (28)	107 (35)	
>30	156 (29)	76 (33)	80 (26)	
Smoking				
Never	251 (46)	114 (47)	137 (45)	0.706
Ex-smoker	223 (41)	94 (39)	129 (42)	
Current	73 (13)	34 (14)	39 (13)	
ASA score				
1	136 (25)	50 (20)	86 (28)	0.003
2	248 (45)	108 (44)	140 (45)	
3	155 (27)	74 (30)	81 (26)	
4	16 (3)	13 (5)	3 (1)	
Preop mGPS				
0	429 (83)	179 (78)	250 (86)	0.007
1	40 (8)	21 (9)	19 (7)	
2	48 (9)	29 (13)	19 (7)	
Tumor site				
Colon	355 (64)	159 (65)	196 (63)	0.658
Rectum	201 (36)	86 (35)	115 (37)	
TNM stage				
0	13 (2)	5 (2)	8 (3)	0.261
I	127 (23)	47 (19)	80 (27)	
II	229 (42)	112 (46)	117 (38)	
III	181 (33)	80 (33)	311 (32)	
Neoadjuvant treatment				
Yes	82 (15)	34 (15)	48 (16)	0.630
No	466 (85)	209 (85)	257 (84)	
Approach				
Open	337 (61)	195 (80)	142 (47)	<0.001
Laparoscopic	212 (39)	49 (20)	163 (53)	
Surgery > 4 h				
Yes	183 (33)	57 (23)	126 (41)	<0.001
No	370 (67)	187 (77)	183 (59)	
Intraoperative transfusion				
Yes	29 (5)	21 (9)	8 (3)	0.002
No	517 (95)	221 (91)	296 (97)	
Stoma				
Yes	164 (30)	72 (29)	92 (30)	0.926
No	390 (70)	173 (71)	217 (70)	
Epidural				
Yes	244 (44)	158 (64)	86 (28)	<0.001
No	308 (56)	87 (36)	221 (72)	
POD 3 CRP				
mg/L [median (range)]	138 (9601)	166 (22–601)	118 (9–430)	<0.001
POD 3 CRP > 150 mg/L				
Yes	239 (45)	136 (57)	103 (35)	<0.001
No	292 (55)	101 (43)	191 (65)	
POD 4 CRP, mg/L [median (range)]	112 (13–528)	118 (13–528)	105 (15–415)	0.018
POD 4 CRP > 150 mg/L				
Yes	153 (33)	79 (36)	74 (31)	0.277
No	308 (67)	142 (64)	166 (69)	
POD 3 albumin				
Median range, g/L	26 (7–40)	25 (14–35)	27 (7–40)	<0.001
POD 3 albumin < 25 g/L				
Yes	189 (36)	104 (44)	85 (19)	0.001
No	332 (64)	130 (56)	202 (81)	
POD 4 albumin				
Median range, g/L	26 (13–35)	25 (14–35)	27 (13–35)	<0.001
POD 4 albumin < 25 g/L				
Yes	170 (37)	97 (44)	73 (31)	0.003
No	285 (63)	121 (56)	164 (69)	
Any complication				
Yes	234 (42)/321	122 (50)	112 (36)	0.001
No	321 (58)	123 (50)	198 (64)	
Infective complications				
Yes	151 (27)	79 (32)	72 (23)	0.021
No	440 (73)	166 (68)	238 (77)	
Anastomotic leak				
Yes	19 (3)	12 (5)	7 (2)	0.103
No	536 (97)	233 (95)	330 (98)	
Clavien–Dindo grade				
0–2	508 (92)	222 (91)	286 (92)	0.540
3–5	47 (8)	23 (9)	24 (8)	
30 day mortality				
Yes	5 (1)	3 (1)	2 (1)	0.659
No	550 (99)	242 (99)	308 (99)	
Adjuvant treatment				
Yes	152 (32)	61 (27)	91 (36)	0.031
No	325 (68)	165 (73)	160 (64)	

*BMI* body mass index, *ASA* American Society of Anesthesiologists, *POD* postoperative day, *CRP* C-reactive protein, *mGPS* preoperative modified Glasgow Prognostic score, *Preop* preoperative

### Impact of Dexamethasone on All Patients

In the unmatched cohort, exceeding the CRP threshold of 150 mg/L on POD 3 was significantly associated with higher rates of any complication (60 vs. 29%, OR 3.60, *p* < 0.001), infective complications (42 vs. 16%, OR 3.87, *p* < 0.001), anastomotic leak (6 vs. 1%, OR 4.16, *p* = 0.011), and Clavien–Dindo grade 3 or higher complications (13 vs. 5%, OR 3.10, *p* = 0.001). In the unmatched cohort (Table 1), 311 patients (56%) received dexamethasone at the induction of anesthesia, of whom 194 received 4 mg and 117 received 8 mg, while 245 (44%) did not. There were significant differences between those patients who received preoperative dexamethasone and those who did not, in ASA grade (*p* = 0.003), preoperative mGPS (*p* = 0.007), laparoscopic surgery (52 vs. 20%, *p* < 0.001), surgery lasting more than 4 h (41 vs. 23%, *p* < 0.001), blood transfusion (3 vs. 9%, *p* = 0.002), and epidural anesthesia (28 vs. 64%, *p* < 0.001). A significantly lower proportion of those who received preoperative dexamethasone exceeded the established CRP threshold of 150 mg/L on POD 3 (33 vs. 55%, *p* < 0.001), but not on day 4. Preoperative dexamethasone was significantly associated with fewer postoperative complications (36 vs. 50%, OR 0.40, *p* = 0.001) and infective complications (23 vs. 32%, OR 0.57, *p* = 0.021), but not anastomotic leak or complication severity.

### Impact of Dexamethasone on the Propensity Score-Matched Cohort

**Fig. 1:**
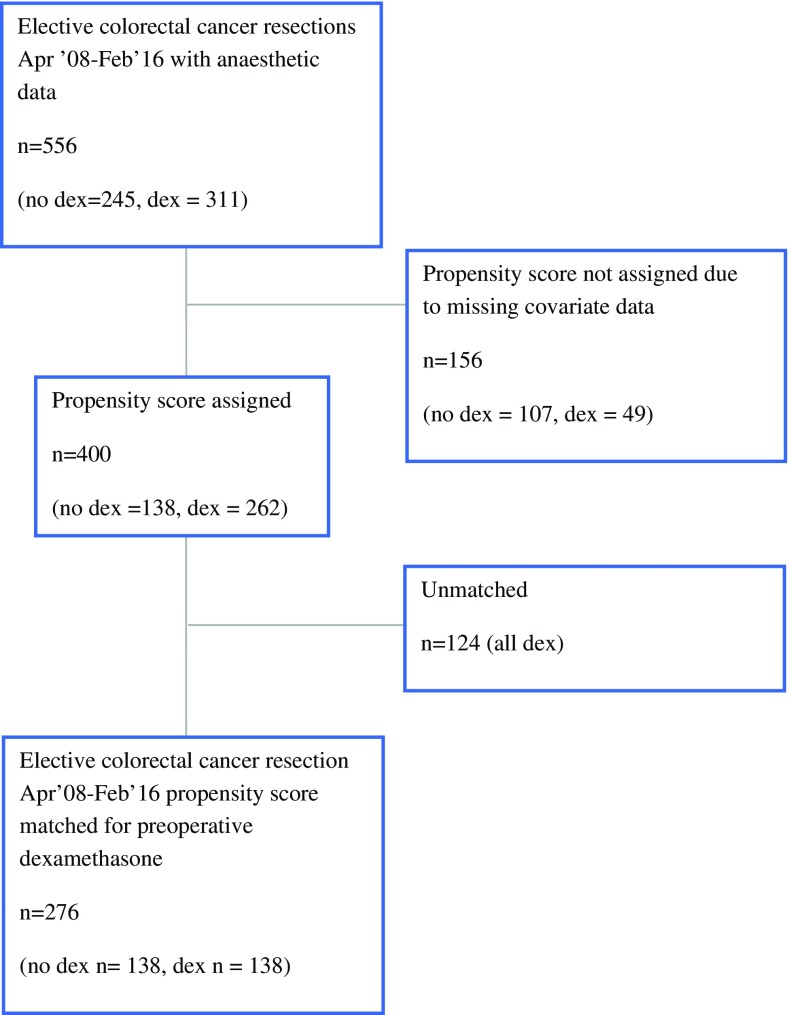
Patient flowchart for preoperative dexamethasone before elective surgery for colorectal cancer. *dex* dexamethasone

**Fig. 2:**
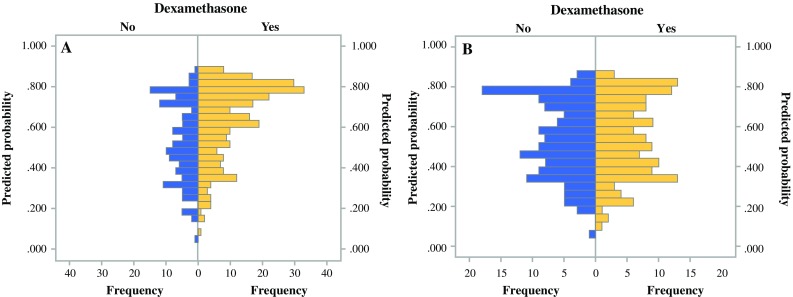
Distribution of propensity scores **a** before (*n* = 400) and **b** after matching (*n* = 276)

Propensity scores could not be assigned to 156 patients due to missing covariate data, leaving 400 patients with propensity scores, of whom 262 had received dexamethasone at the induction of anesthesia and 138 had not (Fig. 1). Overall, 276 patients (138 from each group) were matched based on their propensity score, with a subsequent improvement in the balance of the distribution of propensity scores in each group (Fig. 2).

In the propensity score-matched cohort, exceeding the CRP threshold of 150 mg/L on POD 3 was significantly associated with higher rates of any complication (59 vs. 28%, OR 3.58, *p* < 0.001), infective complications (44 vs. 15%, OR 4.38, *p* < 0.001), and Clavien–Dindo grade 3 or higher complications (13 vs. 6%, OR 2.56, *p* = 0.032), but not anastomotic leak (7 vs. 2%, OR 3.29, *p* = 0.068). Following propensity score matching, the distribution of patient and operative variables was balanced between the two groups (Table 2). A significantly lower proportion of those who received preoperative dexamethasone exceeded the established CRP threshold of 150 mg/L on POD 3 (36 vs. 56%, OR 0.42, *p* = 0.001), but not on day 4. Preoperative dexamethasone was significantly associated with fewer postoperative complications (34 vs. 49%, OR 0.53, *p* = 0.001).

**Table 2:** Association between preoperative dexamethasone and outcomes in propensity score-matched patients undergoing any surgery for colorectal cancer [*n* = 276]

Characteristic	All *n* (%)	Preoperative dexamethasone		*p* value
		No *n* (%)	Yes *n* (%)	
*n*	276 (100)	138 (50)	138 (50)	–
Age, years				
<65	102 (37)	54 (39)	48 (35)	–
65–74	106 (38)	49 (36)	57 (42)	
>74	68 (25)	35 (25)	33 (24)	
Sex				
Male	161 (58)	79 (57)	82 (59)	–
Female	115 (42)	59 (43)	56 (41)	
BMI, kg/m^2^				
<20	16 (6)	8 (6)	8 (6)	–
20–25	97 (35)	54 (39)	43 (31)	
26–30	82 (30)	34 (25)	48 (35)	
>30	81 (29)	42 (30)	39 (28)	
Smoking				
Never	130 (47)	64 (46)	66 (48)	–
Ex-smoker	113 (41)	52 (38)	61 (44)	
Current	33 (12)	11 (8)	11 (8)	
ASA score				
1	72 (26)	36 (26)	36 (26)	–
2	116 (42)	59 (43)	57 (41)	
3	80 (29)	37 (27)	43 (32)	
4	8 (3)	6 (4)	2 (1)	
Preop mGPS				
0	224 (82)	107 (78)	117 (85)	–
1	26 (9)	15 (11)	11 (8)	
2	26 (9)	16 (11)	10 (7)	
Tumor site				
Colon	170 (62)	86 (62)	84 (61)	–
Rectum	10 (38)	52 (38)	54 (39)	
TNM stage				
0	7 (3)	4 (3)	3 (2)	–
I	69 (25)	30 (22)	39 (28)	
II	109 (39)	60 (43)	49 (36)	
III	91 (33)	44 (32)	47 (34)	
Neoadjuvant treatment				
Yes	49 (18)	25 (18)	24 (17)	–
No	227 (82)	113 (82)	114 (83)	
Approach				
Open	184 (67)	93 (67)	91 (66)	–
Laparoscopic	92 (33)	45 (33)	47 (34)	
Surgery > 4 h				
Yes	94 (34)	44 (32)	50 (36)	–
No	182 (66)	94 (68)	88 (64)	
Intraoperative transfusion				
Yes	13 (5)	6 (4)	7 (5)	–
No	263 (95)	132 (96)	131 (95)	
Stoma				
Yes	90 (33)	43 (31)	47 (34)	–
No	186 (67)	95 (69)	91 (66)	
Epidural				
Yes	132 (48)	66 (48)	66 (48)	–
No	144 (52)	72 (52)	72 (52)	
POD 3 CRP				
Median, range, mg/L	143 (17–430)	166 (22–382)	126 (17–430)	<0.001
POD 3 CRP > 150 mg/L				
Yes	123 (45)	75 (56)	48 (35)	0.001
No	145 (55)	58 (44)	87 (65)	
POD 4 CRP				
Median, range, mg/L	121 (13–415)	121 (13–369)	121 (19–415)	0.241
POD 4 CRP > 150 mg/L				
Yes	80 (34)	46 (38)	34 (29)	0.349
No	158 (66)	75 (62)	83 (71)	
POD 3 albumin				
Median, range, mg/L	26 (7–35)	25 (15–35)	26 (7–35)	0.058
POD 3 albumin < 25 g/L				
Yes	96 (37)	52 (40)	44 (33)	0.392
No	166 (63)	78 (60)	88 (67)	
POD 4 albumin				
Median, range, mg/L	26 (14–35)	25 (14–35)	26 (16–35)	0.768
POD 4 albumin < 25 g/L				
Yes	88 (37)	48 (40)	40 (34)	0.749
No	150 (63)	72 (60)	78 (66)	
Any complication				
Yes	115 (42)	68 (49)	47 (34)	0.009
No	161 (58)	70 (51)	91 (66)	
Infective complications				
Yes	78 (28)	45 (33)	33 (24)	0.134
No	198 (72)	93 (67)	105 (76)	
Anastomotic leak				
Yes	13 (5)	9 (7)	4 (3)	0.227
No	263 (95)	129 (93)	134 (97)	
Clavien–Dindo (0–2/3–5)				
Yes	26 (9)	17 (12)	9 (7)	0.152
No	250 (91)	121 (88)	129 (93)	
30 day mortality				
Yes	2 (1)	2 (2)	0 (0)	–
No	274 (99)	136 (98)	138 (100)	
Adjuvant treatment				
Yes	65 (28)	31 (25)	34 (31)	0.728
No	168 (72)	92 (75)	76 (69)	

*BMI* body mass index, *ASA* American Society of Anesthesiologists, *POD* postoperative day, *CRP* C-reactive protein, *mGPS* preoperative modified Glasgow Prognostic score, *Preop* preoperative

### Other Propensity Score Analyses

Analysis of the impact of preoperative dexamethasone on exceeding the POD 3 CRP threshold (Table 3) found a similarly statistically significant probability reduction using regression adjustment (OR 0.53, 95% CI 0.34–0.83), propensity score stratification (OR 0.41, 95% 0.25–0.57), and propensity score matching (0.42, 95% CI 0.26–0.70). The same analysis of the impact of preoperative dexamethasone on postoperative complications (Table 3) found a similarly statistically significant probability reduction using regression adjustment (OR 0.62, 95% CI 0.40–0.96), propensity score stratification (OR 0.62, 95% 0.29–0.95), and propensity score matching (0.53, 95% CI 0.33–0.86).

**Table 3:** Odds ratios for exceeding the CRP threshold of 150 mg/L on POD 3, and postoperative complications with respect to preoperative dexamethasone across the propensity score methods

Propensity score model	*N*	POD 3 CRP > 150 mg/L [OR (95% CI)]	Complication [OR (95% CI)]
Unadjusted	556	0.40 (0.28–0.57)	0.57 (0.41–0.80)
Regression adjustment	400	0.53 (0.34–0.83)	0.62 (0.40–0.96)
Stratification by quintiles (ATE)	400	0.41 (0.25–0.57)	0.62 (0.29–0.95)
Matched 1:1	276	0.42 (0.26–0.70)	0.53 (0.33–0.86)

*POD* postoperative day, *CRP* C-reactive protein, *OR* odds ratio, *CI* confidence interval, *ATE* average treatment effect

### Time-Dependent Effect of Preoperative Dexamethasone

Dexamethasone at the induction of anesthesia had a similar time-dependent effect on postoperative CRP in both the unmatched and matched cohorts. There was a significant reduction in CRP on PODs 1–3, with similar concentrations from POD 4 onward.

## Discussion

The present study reports that dexamethasone, administered at the induction of anesthesia, prior to surgery for colorectal cancer was associated with a reduction in the magnitude of the postoperative SIR and fewer postoperative complications.

Currently, corticosteroids are administered in the perioperative period to reduce postoperative nausea and vomiting.^14^,^15^ However, when taken together with existing evidence,^16^,^17^,^20^ the results of the present study also suggest an important role for reducing the complication rate following surgery for colorectal cancer by attenuating the postoperative stress response. Indeed, the use of preoperative corticosteroids represents a potentially simple and cost-effective method of improving surgical outcomes for a large surgical population. It was of interest that postoperative CRP retained its association with postoperative complications in those patients who had received preoperative dexamethasone. In particular, the CRP threshold of 150 mg/L on POD 3 remained significantly associated with all and infective complications in this group of patients in whom the magnitude of the postoperative SIR was lower as a whole.^21^ Indeed, the results of the present study suggest that the measurement of postoperative CRP in this subgroup remains useful in the clinical setting. For these reasons, the present study in colorectal cancer is timely.

There remain long-standing concerns that corticosteroids may inhibit collagen formation, and therefore wound healing, in the postoperative period. However, neither the present study nor previous meta-analyses have identified a significant negative association with either wound complications or anastomotic leak^14^,^15^ Furthermore, there have been some concerns that preoperative corticosteroids may have a negative impact on oncologic outcome following surgery for colorectal cancer; however, the evidence for this is limited in both numbers and length of follow-up.^22^

The mechanisms by which corticosteroids exert their anti-inflammatory action remain poorly understood. Inhibition of nuclear factor κB (NF-κB) leads to a downregulatory effect on lymphoid tissue and thus adaptive immune responses.^23^ In addition, attenuation of the innate immune response and myeloid tissues occurs as a consequence of reduction of the transcription of proinflammatory cytokines such as IL-6, alongside the inhibition of cyclooxygenase-dependent pathways by increasing the transcription of lipocortins.^15^,^24^,^25^

An important implication of the present and previous results is that postoperative complications are themselves recognized to have a negative impact on oncologic outcomes.^26^ Indeed, the generation of a prometastatic environment through systemic inflammation, as part of the surgical injury and the severity of postoperative complications, has been proposed to promote metastatic disease progression.^27^ Furthermore, it has been proposed that this host response to both the tumor and surgery should become a target for intervention.^28^ Indeed, it may be hypothesized that a reduction in the magnitude of the postoperative SIR, with a consequent reduction in postoperative complication rates, may improve long-term outcomes following surgery for colorectal cancer. Strategies such as the prospective evaluation of perioperative corticosteroids represent a logical starting point.

The main limitation of the present study was its retrospective nature. Significant imbalance between the two groups meant that propensity score matching was used to obtain balanced groups for determination of the treatment effect; however, this resulted in the exclusion of a significant proportion of patients, and does not necessarily help those confounders that are either unmeasured or unknown.^29^ Nonetheless, it was reassuring that the overall treatment effect and its magnitude were similar among the unmatched cohort, the matched cohort, and when propensity regression was applied.^30^ In addition, the nature of the analysis prevented the assessment of any dose–response relationship.

## Conclusions

The results of the present study suggest that the use of preoperative corticosteroids is associated with both attenuation of the magnitude of the SIR and fewer complications following surgery for colorectal cancer. This would suggest that the magnitude of the postoperative SIR and postoperative complications are causally related. Optimal doses and treatment regimens are yet to be determined. Indeed, further prospective randomized trials are necessary before recommendations regarding the use of preoperative dexamethasone in the context of the postoperative SIR can be made.
